# Thoracic gout tophus with abdominal wall protrusion: A case report

**DOI:** 10.1097/MD.0000000000019348

**Published:** 2020-03-06

**Authors:** Yanbing Kao, Zhenyu Wang, Jiali Leng, Zhigang Qu, Xinming Zhuang, Hongyun Ma, Qingxu Song, Zijing Liu, Shuo Sun, Yi Liu

**Affiliations:** a Department of Spinal Surgery; b Department of Hospice, the First Hospital of Jilin University, No. 1, Xinmin St, Chaoyang District, ChangChun City, Jilin Province; c Department of Orthopaedics, Qinghai Provincial People's Hospital, Xining City, Qinghai Province; d Nursing platform of spinal surgery department, the First Hospital of Jilin University, No. 1, Xinmin St, Chaoyang District, ChangChun City, Jilin Province, People's Republic of China.

**Keywords:** abdominal wall weakness, axial gout, dual-energy computed tomography, intercostal nerve

## Abstract

**Rationale:**

A patient presented the abdominal wall protrusion due to tophaceous gout of the spine. Similar cases were not reported in the literature. This study aimed to report a case of tophaceous gout of the spine with abdominal wall protrusion.

**Patient concerns:**

A 38-year-old male patient had a 10-year history of gout and hyperuricemia. He complained of back pain and abdominal wall protrusion.

**Diagnoses:**

The patient was diagnosed with tophaceous gout of the spine with abdominal wall weakness caused by T11 nerve root compression.

**Interventions:**

A semi-lamina decompression was performed at T11-T12. The pathological examination of the specimen demonstrated tophaceous gout of the spine.

**Outcomes:**

After the surgery, the patient's back pain was completely relieved and the abdominal wall weakness significant improved.

**Lessons:**

This case highlighted that axial gout could mimic thoracic disk herniation clinically. The abdominal wall weakness might also be due to single T11 nerve compression by the tophaceous gout of the spine. In patients with a history of gout, axial gout should be considered as one of the differential diagnoses.

## 1 Introduction

Gout is inflammatory arthritis associated with hyperuricemia induced by monosodium urate crystals.^[1]^ The incidence of gout is estimated to be 0.2% to 0.4% worldwide with an annual incidence of 0.01% to 0.015%.^[2]^ When the concentration of uric acid in the blood surpasses the physiological dissolution threshold in humans, the urate crystals deposit in the joints, synovial bursa, and subcutaneous tissue.^[3]^ The mechanism underlying axial gout is unclear and requires a widely accepted large-scale epidemiological survey. However, axial gout with neurological symptoms is rarely reported.

Gout involves all the segments of the spine. Toprover et al reviewed 131 cases of axial gout, showing that gout could occur at any level of the spinal cord. The proportion of lumbar vertebrae, cervical vertebrae, and thoracic spine was 38%, 24.8%, and 17.8%, respectively.^[4]^ Moreover, axial gout could impact any anatomic components of the spine, such as facet joint, vertebral bodies, pedicle, lamina, and ligamentum flavum.^[5]^ Patients had symptoms of spinal stenosis, lumbar radiculopathy, spondylolisthesis, cauda equine syndrome, or spinal infection.

Abdominal wall weakness is rare in the clinic and also confused with abdominal wall hernia. It is caused by the looseness, weakness, or even defect of the myofascial tissue on the abdominal wall, leading to the leakage of the contents of the abdominal cavity. Abdominal wall weakness has been reported to result from intercostal nerve injury caused by surgical procedures or herpes virus infections.^[6–8]^ However, abdominal wall weakness caused by the tophaceous gout of the spine has not yet been reported.

This study reported a rare case of tophaceous gout of the spine with abdominal wall weakness.

## 2 Case description

**Figure 1:**
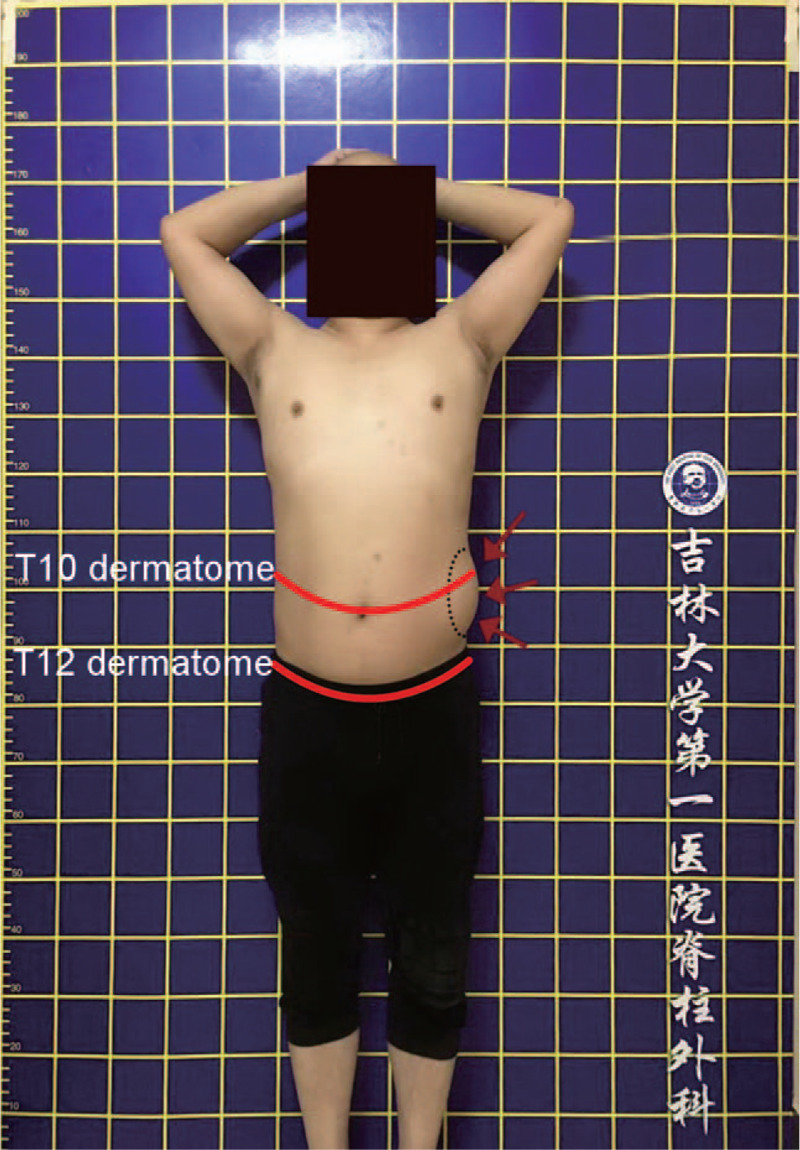
Abdominal wall protrusion appeared between T10 dermatome and T12 dermatome in standing position.

A 38-year-old Chinese male patient had a 2-month history of back and intercostal pain. Following pain for 1 month, a protrusion was detected in the lower left abdomen of the patient when standing (Fig. 1), which disappeared in the lateral decubitus position. The patient was once misdiagnosed with abdominal wall hernia. However, computed tomography (CT) and abdominal color ultrasound did not support this diagnosis. The examination results showed that the bilateral abdominal wall muscle was continuous without breakage. The patient visited our hospital for a distinct diagnosis and further treatment. The medical records revealed that the patient had a 10-year history of gout and hyperuricemia but did not undergo standard and systemic gout treatment. Tophaceous deposits were present in the hands and toes for at least 5 years. Furthermore, the patient had high alcohol intake for 15 years. When he suffered gouty attacks, clindamycin and dexamethasone were given to relieve pain.

The physical examination exhibited a mild pain to percussion on T11–T12 spinous processes, but radiating pain was not evident. The numbness was experienced in the left T11–T12 intercostal area and the related lateral abdominal wall. Neurological examinations revealed normal sensory and motor functions of the lower limbs. Patients had no abnormal reflexes, and pathological reflexes were negative in both legs. Also, a decreased left lower abdominal reflex was found. Bilateral cremasteric reflexes were normal. The patient's largest abdominal circumference across the core of the protrusion was 110 cm. The abdominal wall protrusion range was 12 cm (longitudinal diameter) × 20 cm (latitudinal diameter). The laboratory values were as follows:

Uric acid, 701 μmol/L; Erythrocyte sedimentation rate, 14 mm/1 h; C-reactive protein, 11 mg/L.

**Figure 2:**
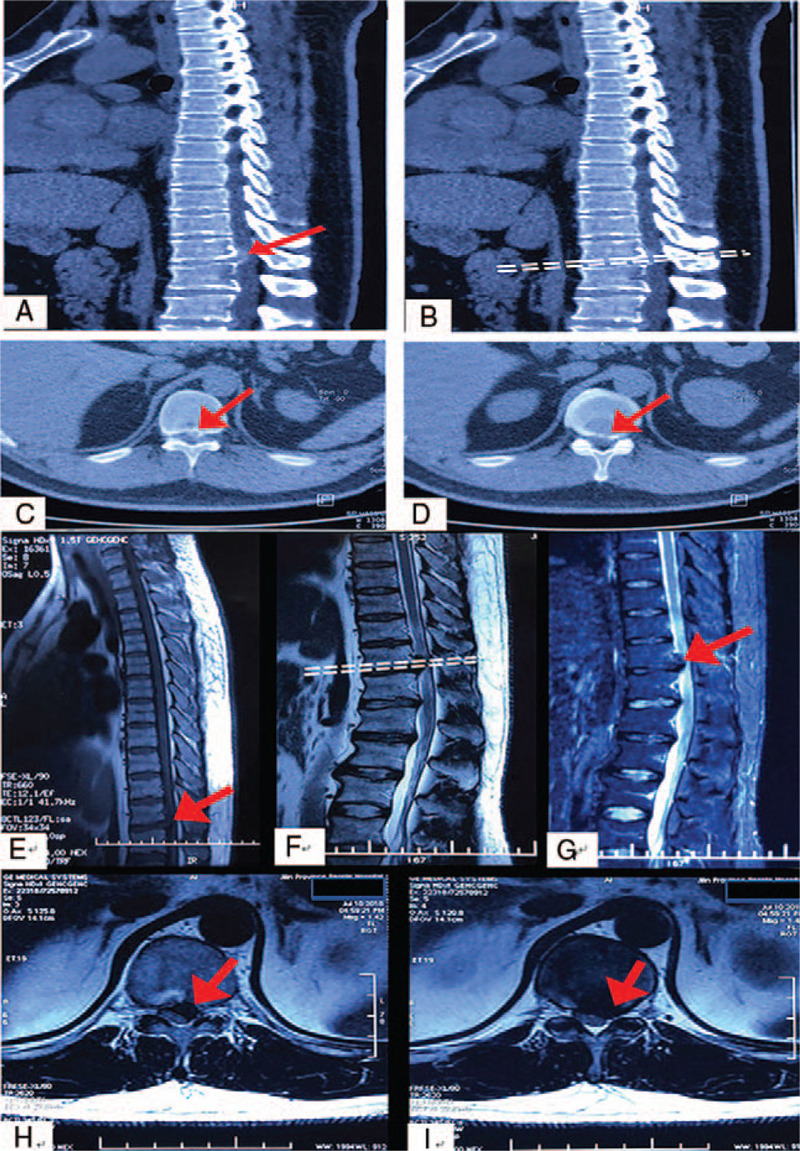
Preoperative CT and MRI images: (A) Sagittal plane showed calcification surrounding the herniated disk at T11/T12 levels. (B) Positioning phase. (C and D) Axial plane showed disk herniation and spinal stenosis at T11/T12 levels. (E) T1-weighted image shows the lesions at T11/T12 levels. (F) T2-weighted image shows the lesions at T11/T12 levels. (G) T2 fat suppression images shows the lesions at T11/T12 levels. (H and I) Axial images show the lesion is closed to spinal cord and enters the nerve root canal.

The CT of the thoracic spine revealed disk herniation and spinal stenosis at T11/T12 levels. The sagittal and axial planes of thoracic CT showed a high-density shadow surrounding the herniated disk (Fig. 2), which was considered as disk calcification on the magnetic resonance imaging (MRI) of the thoracic spine. The disks of T11/T12 showed posterior abdominal wall weakness. Furthermore, thoracic disk degeneration was evident at the level of T9/T10, T10/T11, and T11/T12.

**Figure 3:**
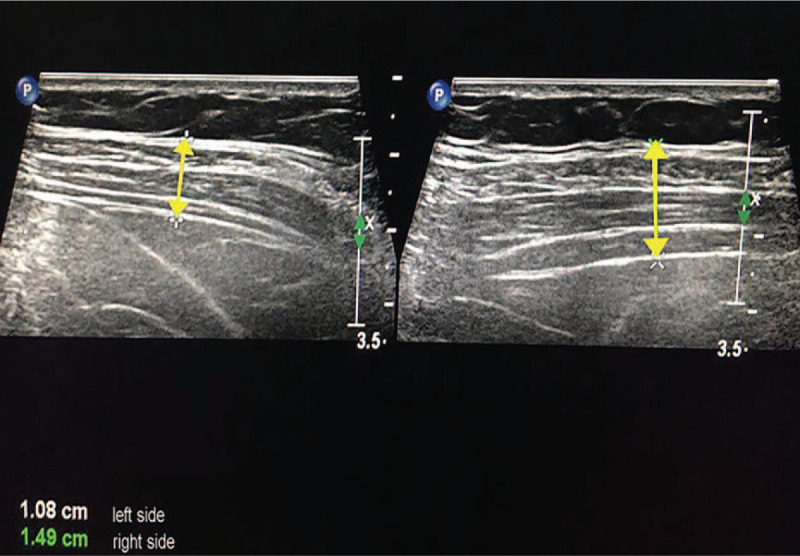
Preoperative color Doppler ultrasound: The image showed that the left anterolateral abdominal wall was thinner than the right anterolateral abdominal wall.

MRI revealed abnormal hyperintensity in the disk on the axial plane (Fig. 2). In addition, the abdominal color Doppler ultrasound in the standing position showed that the left anterolateral abdominal wall was thinner than the right anterolateral abdominal wall (Fig. 3). The patient was diagnosed with thoracic disk herniation with abdominal wall weakness based on the symptoms and imaging examination. Posterior thoracic decompression, discectomy, and internal fixation were performed to achieve neurological decompression, improve abdominal wall weakness, and relieve back and intercostal pain.

**Figure 4:**
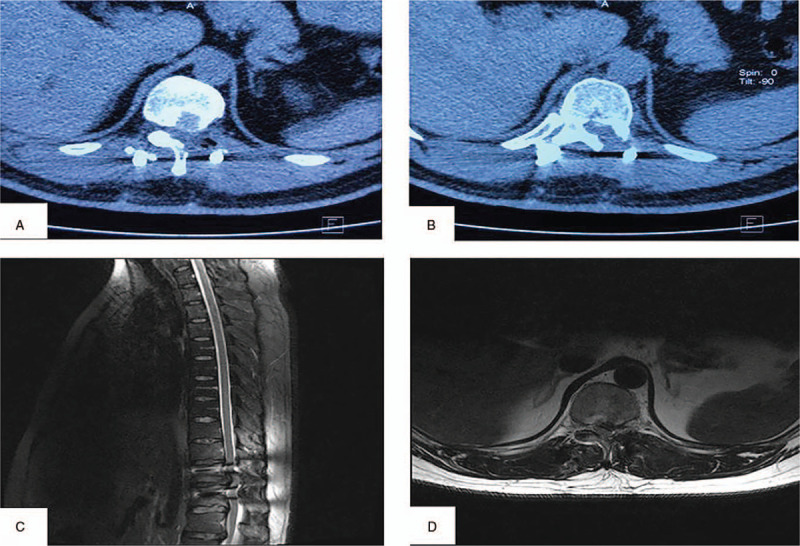
Postoperative CT images and MRI images: (A and B) Postoperative CT images showed adequate semi-lamina decompression at T11/T12 levels. (C and D) Postoperative MRI images showed adequate semi-lamina decompression at T11/T12 levels.

During the surgery, the left articular process and lamina were removed. A mass with a complete capsule was found on exploring the suspected thoracic disk herniation. After the capsule was opened, a white, silt-like, and granular crystal was observed, removed, and prepared for the pathological examination. Combined with the history of gout, the original diagnosis of thoracic disk herniation was negated and an intraoperative diagnosis of the tophaceous gout of the spine was made. Then, the left nerve root canal was decompressed, and the dorsal and ventral sides of the spinal cord were explored. Discectomy was not conducted because disk herniation was not found. Furthermore, due to the tight adhesion between the frontal mass and the dura mater, the complete resection of the mass capsule was aborted after the effective decompression of the left nerve root, to prevent spinal cord injury, dura tear, and cerebrospinal fluid leakage. Consequently, a tiny residue of the mass capsule was left on the ventral side of the spinal cord. However, decompression of the spinal cord and spinal nerve was achieved after the resection. A pedicle screw system was applied to prevent segmental instability. A pathological examination showed that the deposits of monosodium urate crystals were surrounded by multinucleated giant cells and monocytes. The pathological examination is consistent with our intraoperative diagnosis. According to pathological result, symptoms, laboratory value and imaging data, the final diagnosis was tophaceous gout of thoracic spine. After the surgery, his back pain and numbness were significantly and immediately relieved. The patient had a normal gait 3 days after the surgery. However, the left abdominal wall protrusion in the standing position did not disappear immediately. The back pain and numbness disappeared during the first follow-up 3 months after the surgery. The range of the abdominal wall protrusion reduced to 12 cm (longitudinal diameter) × 18 cm (latitudinal diameter). During the second follow-up 6 months after the surgery, the patient underwent CT and MRI. MRI did not show any compression around the spinal cord and nerve root (Fig. 4). The thoracic CT also revealed that the calcification surrounding the tophaceous gout was removed, and no high-density shadow was detected in the spinal canal (Fig. 4). The range of the abdominal wall protrusion reduced to 11 cm (longitudinal diameter) × 18 cm (latitudinal diameter). Additionally, the abdominal circumference of the patient reduced to 106 cm. The abdominal color Doppler ultrasound showed that the left anterolateral abdominal wall was thicker than the preoperative thickness by 2 mm.

## 3 Discussion

This study reported a rare case of axial gout with tophaceous deposits in the thoracic spinal canal. The patient had severe pain and left abdominal wall thickness. A total of 23 cases of the tophaceous gout of the spine have been reported to date (Table 1).^[2,9–30]^. The clinical manifestations were not consistent; the decrease in muscle power and positive pathological signs were the most common characteristics (Table 1). However, the tophaceous gout of the spine with abdominal wall weakness was not reported previously.

**Table 1:**
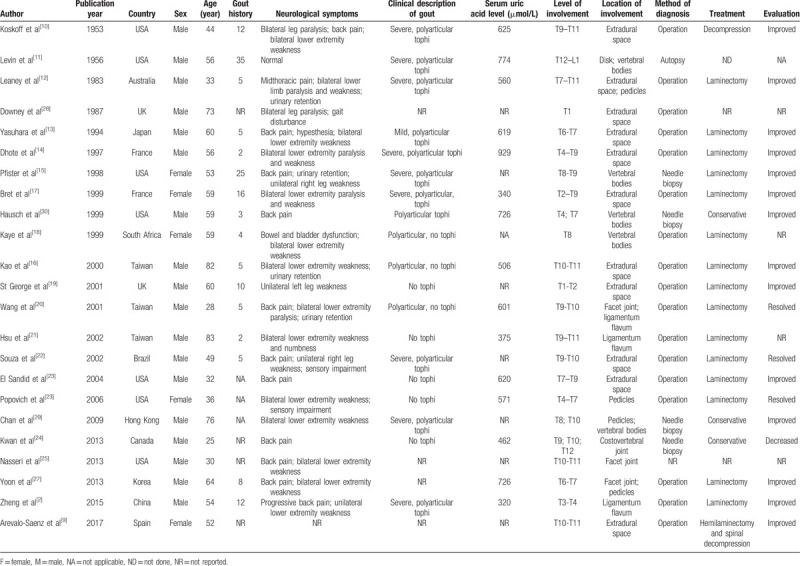
Demographic and clinical characteristics of patients with axial gout.

Typically, the ventral rami of the inferior six thoracic nerves (T7–T12)—also known as the intercostal or thoracoabdominal nerves—contribute to the innervation of all muscles of the anterolateral abdominal wall to varying degrees.^[31]^ Fahim et al performed 32 cadaveric dissections to isolate the individual muscle layers and nerve supply. This anatomical study revealed a limited insertion of the T9 and T10 nerves into the anterolateral abdominal muscles. The most significant intercostal nerve contributions to this muscle were from the T11/T12 segment.^[31]^ Intriguingly, these anatomical studies might explain the cause of abdominal wall weakness in the present case; the T11 thoracic nerve root was compressed by tophaceous gout. The weakness was primarily located in the anterolateral abdominal wall between the umbilicus and the inguinal ligament, which is mainly in T11 dermatome (Fig. 1). The number of patients diagnosed with the tophaceous gout of the spine is extremely low. The present case involved the T11/T12 segment, and the clinical manifestations were associated with spinal cord compression without T11 or T12 thoracic nerve root compression. This could explain why the tophaceous gout of the spine with abdominal wall weakness has not yet been reported.

Interestingly, the abdominal wall weakness is reported as one of the postoperative complications after abdominal surgery.^[32]^ Typically, the iatrogenic intercostal nerve injury is the most common cause of abdominal wall weakness.^[6–8]^ However, single nerve root compression is not considered as the cause of abdominal wall weakness because it results from the compression of a single thoracic nerve root, which has not been reported previously. However, the present case showed that single thoracic root compression resulted in abdominal wall weakness. Therefore, single T11 thoracic root compression should be listed as one of the putative causes of abdominal wall weakness.

In the present case, the neurological symptoms caused by T11 nerve root compression included no spinal cord compression. Therefore, hemilaminectomy was performed, and the nerve root was fully decompressed. This relieved the pain, and the abdominal wall weakness improved significantly. Early and adequate decompression might have benefited neurological recovery. It also revealed that the anterolateral abdominal wall recovered after decompression.

The present case showed that the tophaceous gout of the spine appeared as homogeneous areas of intermediate-to-low signal intensity on T1-weighted images. Homogeneous hypointensity was found on T2-weighted images. This hypointensity might be due to immobile protons in tophaceous gout, such as regions of calcifications, mature fibrous tissue, or hemosiderin deposition.^[2]^ The tophaceous gout is similar to a degenerated disk on T2-weighted images. Thus, MRI failed to differentiate tophaceous gout from thoracic disk herniation. However, the tophaceous gout of the spine can also appear as areas of hyperintensity on T2-weighted images, as reported previously.^[2]^ The commonly used imaging modalities can help in the diagnosis of axial gout, but they are not specific for detecting the deposition of urate crystals.^[33]^ Dual-energy CT (DECT) emerges as a promising, sensitive, and specific imaging modality for the identification and volumetric quantification of tophaceous gout.^[33]^ The identification of urate deposition using DECT has already been included as one of the diagnostic criteria of gout.^[34]^ Nonetheless, most of the published studies on DECT are based on patients with axial gout in the peripheral joints.

However, the mechanism underlying axial gout is unclear and requires a widely accepted large-scale epidemiological survey. Konatalapalli et al performed a cross-sectional study on axial gout, in which 17/48 (35%) participants had the CT evidence of spinal gout and 7/48 (15%) had spinal tophi.^[35]^ However, the exact incidence of tophaceous gout of the spine might be much higher than that detected. Also, axial gout should be a crucial differential diagnosis based on the gout history and neurological symptoms of patients. DECT may be recommended besides routine spinal MRI for patients suspected of having axial gout. A limitation of this study was the relatively short follow-up time, which should be increased to achieve better outcomes regarding the patient's nerve and muscle function.

## 4 Conclusion

This case highlighted that axial gout could mimic thoracic disk herniation clinically. The abdominal wall weakness might also be due to single T11 nerve compression by the tophaceous gout of the spine. In patients with a history of gout, axial gout should be considered as one of the differential diagnoses.

## Author contributions

**Investigation**: Zhigang Qu, Xinmin Zhuang, Qingxu Song, Zijing Liu, Hongyun Ma, and Shuo Sun

**Writing – original draft**: Jiali Leng and Yanbing Kao

**Writing – review & editing**: Zhenyu Wang and Yi Liu

## References

[1] Doherty M New insights into the epidemiology of gout. Rheumatology 2009;48:2–8. 19447779 10.1093/rheumatology/kep086

[2] Zheng ZF Shi HL Xing Y Thoracic cord compression due to ligamentum flavum gouty tophus: a case report and literature review. Spinal Cord 2015;53:881–6. 26078231 10.1038/sc.2015.93 PMC5399141

[3] Ding Y Wang W Jiang W Tophaceous gout causing thoracic spinal cord compression: case report and review of the literature. Neuro-Chirurgie 2018;64:171–6. 29731313 10.1016/j.neuchi.2017.11.002

[4] Toprover M Krasnokutsky S Pillinger MH Gout in the Spine: Imaging, Diagnosis, and Outcomes. Curr Rheumatol Rep. [Article]. 2015 Dec;17:9 10.1007/s11926-015-0547-7 26490179

[5] Hou LC Hsu AR Veeravagu A Spinal gout in a renal transplant patient: a case report and literature review. Surg Neurol 2007;67:65–73. discussion 73. 17210304 10.1016/j.surneu.2006.03.038

[6] Quraishi NA Konig M Booker SJ Access related complications in anterior lumbar surgery performed by spinal surgeons. Eur Spine J 2013;22: Suppl 1: S16–20. Suppl 1. 23250515 10.1007/s00586-012-2616-1 PMC3578511

[7] Gardner GP Josephs LG Rosca M The retroperitoneal incision - an evaluation of postoperative flank bulge. Arch Surg 1994;129:753–6. 8024457 10.1001/archsurg.1994.01420310085015

[8] Ohno S Togawa Y Chiku T Postherpetic pseudohernia: delayed onset of paresis of abdominal muscles due to herpes zoster causing an ipsilateral abdominal bulge. BMJ Case Rep 2016;2016. 10.1136/bcr-2016-215377 PMC4885417 27229900

[9] Arevalo-Saenz A Gonzalez-Alvaro I Pulido-Rivas P Medullar thoracic compression by tophaceous gout: presentation of a case and review of the literature. Rev Neurol 2017;65:368–72. 28990647

[10] Koskoff YD Morris LE Luic LG Paraplegia as a complication of gout. JAMA 1953;152:37–8. 10.1001/jama.1953.63690010013007h 13034524

[11] Levin MH Lichtenstein LS Scott##HW Pathologic changes in gout: survey of eleven necropsied cases. Am J Pathol 1956;32:871–95. 13354714 PMC1942635

[12] Leaney BJ Calvert JM Tophaceous gout producing spinal cord compression. J Neurosurg 1983;58:580–2. 6827352 10.3171/jns.1983.58.4.0580

[13] Yasuhara K Tomita Y Takayama A Fujikawa H Otake Y Takahashi K Thoracic myelopathy due to compression by the epidural tophus: a case report. Journal of spinal disorders. [Case Reports;]. 1994;7:82–5 10.1097/00002517-199407010-00012 8186594

[14] Dhote R Roux FX Bachmeyer C Extradural spinal tophaceous gout: evolution with medical treatment. Clin Exp Rheumatol 1997;15:421–3. 9272305

[15] Pfister AK Schlarb CA O’Neal JF Vertebral erosion, paraplegia, and spinal gout. Am J Roentgenol 1998;171:1430–1. 9798898 10.2214/ajr.171.5.9798898

[16] Kao MC Huang SC Chiu CT Thoracic cord compression due to gout: a case report and literature review. J Formos Med Assoc 2000;99:572–5. 10925570

[17] Bret P Ricci AC Saint-Pierre G Thoracic spinal cord compression by a gouty tophus. Case report. Review of the literature. Neuro-Chirurgie 1999;45:402–6. 10717591

[18] Kaye PV Dreyer MD Spinal gout: an unusual clinical and cytological presentation. Cytopathology 1999;10:411–4. 10607012 10.1046/j.1365-2303.1999.00195.x

[19] St George E Hillier CEM Hatfield R Spinal cord compression: an unusual neurological complication of gout. Rheumatology 2001;40:711–2. 11426037 10.1093/rheumatology/40.6.711

[20] Wang LC Hung YC Lee EJ Acute paraplegia in a patient with spinal tophi: a case report. J Formos Med Assoc 2001;100:205–8. 11393117

[21] Hsu CY Shih TTF Huang KM Tophaceous gout of the spine: MR imaging features. Clin Radiol 2002;57:919–25. 12413917 10.1053/crad.2001.1001

[22] Souza AWS Fontenele S Carrete H Involvement of the thoracic spine in tophaceous gout. A case report. Clin Exp Rheumatol 2002;20:228–30. 12051405

[23] Popovich TCJ Rai AT Carson LV Spinal cord compression by tophaceous gout with fluorodeoxyglucose-positron-emission tomographic/MR fusion imaging. Am J Neuroradiol 2006;27:1201–3. 16775264 PMC8133925

[24] Kwan BYM Osman S Barra L Spinal gout in a young patient with involvement of thoracic, lumbar and sacroiliac regions. Joint Bone Spine 2013;80:667–8. 23566660 10.1016/j.jbspin.2013.02.012

[25] Nasseri F Myers A Shah K Moron FE Severe back pain and lower extremities weakness in a young male. British Journal Of Radiology. [Article]. 2013;86:4. 10.1259/bjr.20110685 PMC3635793 23537521

[26] El Sandid M, Ta H. Another presentation of gout. Annals of internal medicine. [Case Reports; Letter]. 2004;140:W32. 10.7326/0003-4819-140-8-200404200-00037-w2 15096364

[27] Yoon J-W Park K-B Park H Tophaceous gout of the spine causing neural compression. Korean J Spine 2013;10:185–8. 24757485 10.14245/kjs.2013.10.3.185 PMC3941767

[28] Downey PR Brophy B Sage MR Four unusual cases of spinal cord compression. Australas Radiol 1987;31:136–41. 3632520 10.1111/j.1440-1673.1987.tb01799.x

[29] Chan AT Leung JL Sy AN Thoracic spinal gout mimicking metastasis. Hong Kong Med J 2009;15:143–5. 2009. 19342742

[30] Hausch R Wilkerson M Singh E Tophaceous gout of the thoracic spine presenting as back pain and fever. J Clin Rheumatol 1999;5:335–41. 19078426 10.1097/00124743-199912000-00007

[31] Fahim DK Kim SD Cho D Avoiding abdominal flank bulge after anterolateral approaches to the thoracolumbar spine: cadaveric study and electrophysiological investigation. J Neurosurg Spine 2011;15:532–40. 21819186 10.3171/2011.7.SPINE10887

[32] Hoffman RS Smink DS Noone RB Surgical repair of the abdominal bulge: correction of a complication of the flank incision for retroperitoneal surgery. J Am Coll Surg 2004;199:830–5. 15501128 10.1016/j.jamcollsurg.2004.07.009

[33] Ng W Sin CH Wong CH Unusual presentation of spinal gout: 2 cases report and literature review. J Orthop Case Rep 2017;7:50–4. 10.13107/jocr.2250-0685.946 PMC5868884 29600211

[34] Neogi T Jansen T Dalbeth N Gout classification criteria: an American College of Rheumatology/European League against Rheumatism collaborative initiative (vol 74, pg 1789, 2015). Ann Rheum Dis 2016;75:473–573. 26359487 10.1136/annrheumdis-2015-208237 PMC4602275

[35] Konatalapalli RM Lumezanu E Jelinek JS Correlates of axial gout: a cross-sectional study. J Rheumatol 2012;39:1445–9. 22505703 10.3899/jrheum.111517

